# A System of Human Anatomy, General and Special

**Published:** 1844-09

**Authors:** 


### Bibliographical Notices.

*A System of Human Anatomy, General and Special.* By Erasmus Wilson, M. D., Lecturer on Anatomy, London. Second American edition, by Paul Goddard, A. M., M. D., &c., with over two hundred illustrations, by Gilbert. From the second London edition. Philadelphia: Lea & Blanchard, 1844. pp. 607

This work is too well known to require much commendation from us. Had we time, however, we should speak of it more at length, but as we are now just closing the matter for the present number of our Journal, we can only annex the following notice from a cotemporary Journal.

"It might seem to the unreflecting, that the different organs of the human body being fixed in their position and arrangement, and well described in existing works, no new publication on anatomy would be called for—but this is an error. Within the present century the modes of anatomical investigation have been materially modified, new views have arisen, and the grouping together of the various divisions has experienced a corresponding modification. The plan, too, of illustrations by wood cuts, has been introduced, so that the student not only reads the description of an organ, but has it graphically represented before him. The work before us contains every thing to entitle it to adoption by the medical student. The author's plan is excellent, and the execution all that could be desired. It simplifies an important branch of scientific inquiry as far as practicable; is throughout accurate, and the English edition, which has been remarkably successful, is rendered still more valuable by the additions of that competent anatomist, Dr. Goddard. We would not disparage the claims of other anatomical works which are before the public, yet we must conscientiously say that we know of none that is more essential to every anatomical student than the one before us, and we are glad to see by the rapid appearance of a second edition, that our views in regard to it are endorsed by the general approbation of the profession."

